# Disseminated African Histoplasmosis in an Immunocompetent Patient in Morocco: A Case Report

**DOI:** 10.7759/cureus.77668

**Published:** 2025-01-19

**Authors:** Lamis El Yamani, Khadija Kaddar, Kaoutar Belharti, Nada Zizi, Siham Dikhaye

**Affiliations:** 1 Dermatology, Mohammed VI University Hospital, Oujda, MAR

**Keywords:** disseminated histoplasmosis, endogenous reactivation, immunocompetent, itraconazole, mycology

## Abstract

Histoplasmosis, caused by the fungus *Histoplasma capsulatum*, presents in two varieties: American histoplasmosis and African histoplasmosis. We report an unusual presentation of disseminated histoplasmosis in a 22-year-old immunocompetent African patient residing in Morocco, a non-endemic country. The patient was originally from Togo (the town of Kara), which he had not visited for three years. Symptoms included a diffuse rash, coxalgia, and hepatosplenomegaly. The patient likely experienced endogenous reactivation of a latent infection acquired in Togo where histoplasmosis is endemic. Diagnosis involved a skin biopsy revealing *Histoplasma capsulatum*, and treatment with oral itraconazole showed positive outcomes. Unlike American histoplasmosis, the association of African histoplasmosis with HIV is undetermined. The article underscores the importance of considering histoplasmosis in diverse clinical presentations, even in non-endemic regions, emphasizing early diagnosis and appropriate treatment for improved outcomes.

## Introduction

Histoplasmosis is a deep mycosis caused by the fungus *Histoplasma capsulatum*. This disease can manifest in various forms, from asymptomatic to severe disseminated infection [1]. There are two varieties of *H. capsulatum*: the American histoplasmosis found worldwide and more prevalent in the Americas and the African found primarily in Africa [1].

Histoplasmosis is typically acquired through the inhalation of fungal spores from contaminated soil or guano [1]. In the United States, histoplasmosis is most commonly associated with pulmonary infections and is particularly prevalent among individuals with compromised immune systems, such as those with human immunodeficiency virus (HIV). African histoplasmosis, however, more frequently involves the skin and bones and is less commonly linked with HIV infection. The data on incidence are limited in Africa despite the significant burden of HIV [2].

Progressive disseminated histoplasmosis is a severe form of the disease that occurs when the infection spreads beyond the lungs to other organs. This condition is often seen in immunocompromised patients but can rarely affect immunocompetent individuals, particularly following high-level exposure to the pathogen or reactivation of a latent infection [3]. We present a unique case of disseminated African histoplasmosis in a 22-year-old immunocompetent patient residing in Morocco, where histoplasmosis is not endemic. This case underscores the importance of considering histoplasmosis in the differential diagnosis of patients with compatible symptoms, even in non-endemic regions, and highlights the need for early diagnosis and appropriate treatment.

## Case presentation

A 22-year-old African patient consulted the dermatology department for a diffuse skin rash associated with coxalgia evolving four months before his admission. He had a history of malaria infection for which he was treated at the age of 10 years. The patient also reported frequent contact with bats in Togo. Currently residing in Morocco, the patient is originally from Togo (the town of Kara), and his last visit to Togo was three years before the onset of the rash.

The patient appeared well nourished and in no distress, and his body temperature was 37.1°C with a heart rate of 82 bpm and a respiratory rate of 15 cpm. The blood pressure was 120/70 mmHg and oxygen saturation was 98% on room air.

**Figure 1:**
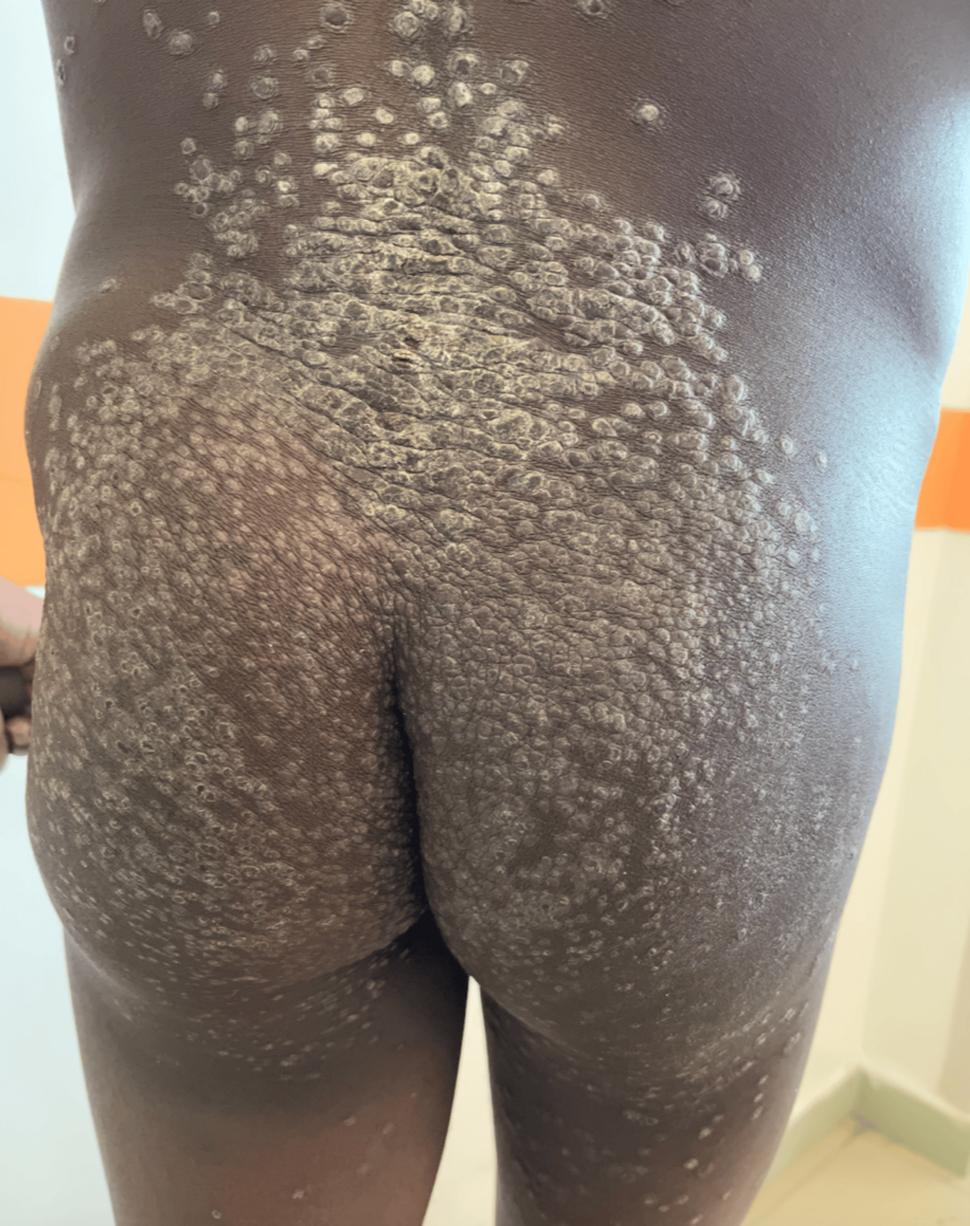
Cutaneous histoplasmosis. The figure shows hyperkeratotic lesions characteristic of psoriasiform histoplasmosis on the patient's buttocks. The lesions are firm papules, some with central umbilication and others with hyperkeratotic surfaces. Clinical significance: These skin lesions are indicative of disseminated histoplasmosis and are key in differentiating it from other dermatological conditions. Location and distribution: The lesions are distributed over the back, buttocks, trunk, limbs, face, scalp, axillae, and pubis.

Dermatologic examination revealed multiple firm papules, some of which had central umbilication (molluscum-like papules) while others had hyperkeratotic lesions over the back, buttocks, trunk, limbs, face, scalp, axillae, and pubis (Figure 1).

During the osteoarticular evaluation, the patient expressed pain on palpation of the right hip joint with limitations of mobility. The patient had enlarged and tender bilateral inguinal lymph nodes, hepatomegaly, and splenomegaly. The pulmonary examination was normal.

Given the presence of diffuse umbilicated papules all over the skin associated with bone pain, hepatosplenic and lymph node involvement, as well as the patient's geographical origin, the diagnosis of African histoplasmosis was strongly suspected. The main differential diagnoses were other deep mycoses such as cryptococcosis, penicilliosis, and blastomycosis. Hip pain, as well as hepatosplenic and lymph node involvement, were consistent with disseminated disease.

Laboratory results demonstrated normal blood count and liver and kidney function tests. C-reactive protein level was 65 mg/L, and protein electrophoresis showed hypoalbuminemia associated with polyclonal hypergammaglobulinemia correlating with the biological inflammatory syndrome. The skin biopsy showed psoriasiform spongiotic dermatosis associated with multiple histiocytes revealing intracellular organisms consistent with *Histoplasma capsulatum*. The diagnosis of histoplasmosis was confirmed by pathological analysis of skin samples, which ruled out other differential diagnoses.

**Figure 2:**
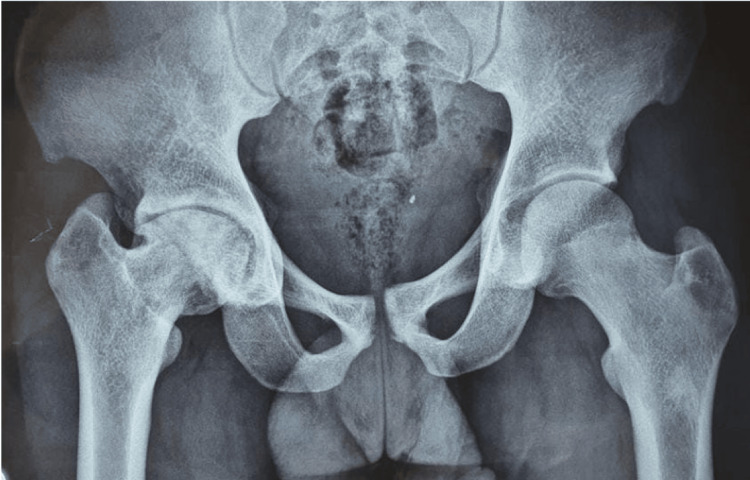
Femoral head osteonecrosis. X-ray showing the loss of sphericity of the femoral head and the loss of joint line regularity associated with subchondral condensation consistent with femoral head osteonecrosis.

The hip X-ray and MRI showed the loss of sphericity of the femoral head and the loss of joint line regularity associated with subchondral condensation consistent with femoral head osteonecrosis (Figure 2).

Chest X-ray was unremarkable and ultrasonography of the abdomen and pelvis showed hepatomegaly associated with splenomegaly secondary to stimulation of the reticuloendothelial system by the infection in the context of a disseminated infection. The patient also underwent a workup for immune deficiency, which was normal. Serologic tests for HIV were negative and lymphocyte typing was normal.

The patient was diagnosed with progressive disseminated histoplasmosis on the basis of clinical findings, notably the characteristic cutaneous rash associated with bone involvement, as well as dissemination of the infection to the reticuloendothelial system characterized by hepatomegaly and splenomegaly, and histopathological confirmation of the infection.

**Figure 3:**
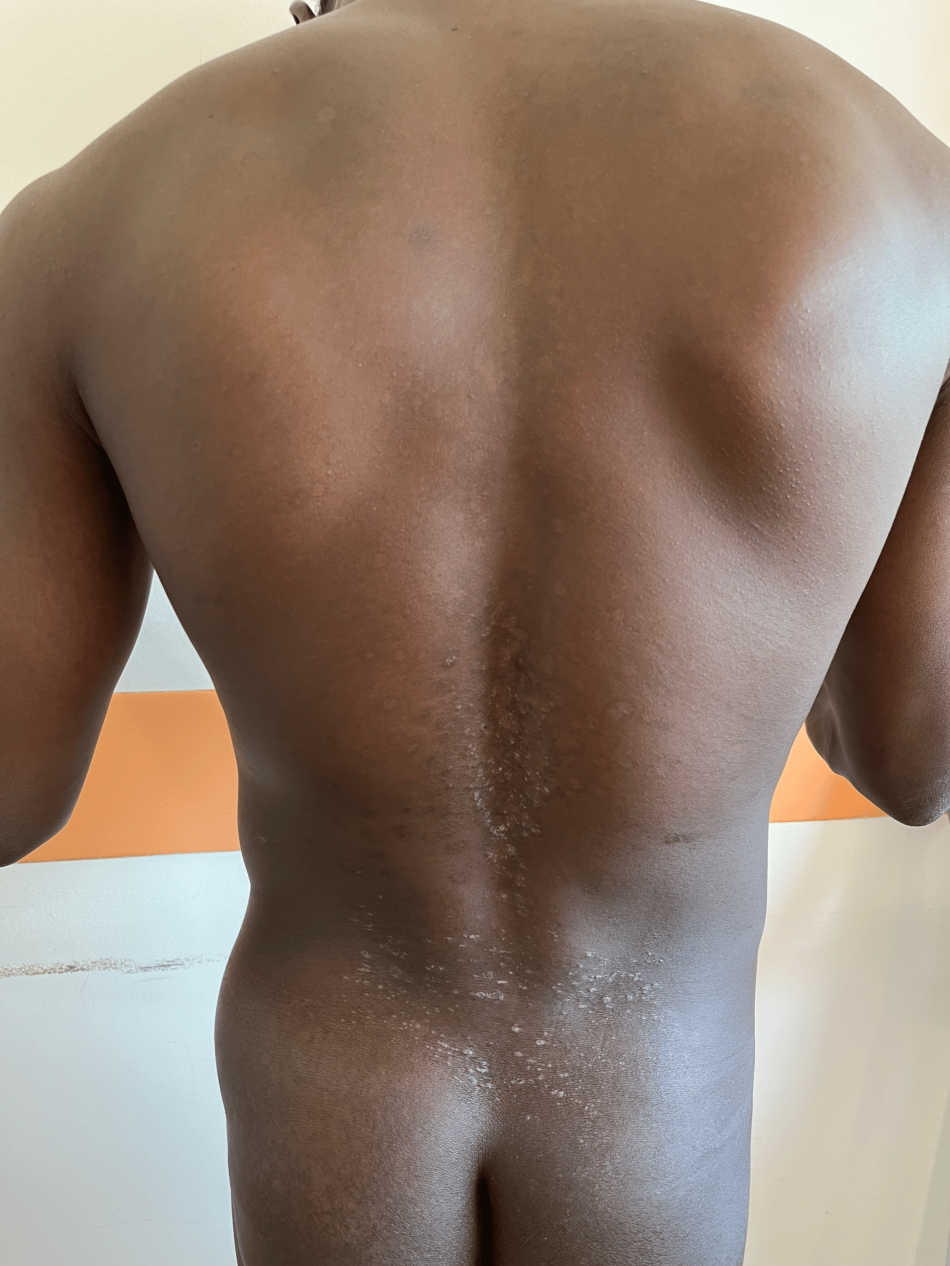
Evolution of cutaneous rash after one month's treatment with itraconazole. After one month of itraconazole, the molluscum-like papules and hyperkeratotic lesions over the back and buttocks started to resolve.

The patient was started on oral itraconazole 200 mg twice daily for 12 months. Conservative treatment of hip osteonecrosis by traction and physiotherapy was undertaken to avoid further deterioration of the affected hip. After one month of itraconazole, the diffuse rash noted on admission began to resolve with no recurrence after one year of follow-up (Figure 3).

## Discussion

Histoplasmosis is a deep mycosis that is caused by the fungus *Histoplasma capsulatum*. There are two varieties of *Histoplasma capsulatum* that infect humans: *Histoplasma capsulatum var. capsulatum*, which represents the American variant, and *Histoplasma capsulatum var. duboisii*, which represents the African variant [1].

The American histoplasmosis is found all over the world, particularly in North and Central America, while *H. capsulatum. duboisii* only exists in Africa [1].

Histoplasma capsulatum is transmitted through the inhalation of conidia from the soil, and exposure to nitrogen-rich guano has been proven to increase the risk of transmission of histoplasmosis [3,4]. Thus, the transmission of the infection may occur through multiple activities such as cave exploration or exposure to bird roosts or poultry that cause the inhalation of the fungus [3,4].

Infection usually occurs shortly after exposure to the pathogen, but it can also occur in people living in non-endemic areas; this has been explained by endogenous reactivation of latent infection acquired during travel or a previous stay in the endemic zone [5]. In fact, fungi have the ability to stay inactive for years after the initial infection has resolved. Typically, this dormancy does not cause clinical issues unless the individual later becomes immunocompromised. In such cases, the body's T-cell-mediated immunity may no longer be able to control the fungi, leading to the reactivation of the infection. Those most at risk for reactivation include individuals with HIV, those treated with tumor necrosis factor inhibitors, and recipients of solid organ transplants. Thus, the reactivation risk must be taken into account in clinical practice [6]. This is the case of our patient who likely experienced an endogenous reactivation of latent infection since his last visit to Togo was three years before the onset of the symptoms; furthermore, histoplasmosis infection seldom occurs in Morocco [7] whereas it is endemic to Togo where incidence is estimated at 330 cases per year [8], the majority of which take place in rural areas where the health infrastructure is poor [8]. The severity of the disease depends on the immune status of the patient and the amount of inoculum exposure [5]. In immunocompromised hosts, it can cause a potentially fatal disseminated disease [1].

American histoplasmosis mainly affects the lungs, while African histoplasmosis mainly affects the skin and skeleton; however, the African form can also be responsible for a disseminated form [9], which is the case of our patient who presented cutaneous, skeletal, splenic, and hepatic histoplasmosis. The peculiarity in our case resides in the dissemination of lesions in a non-HIV-infected patient without any other form of immunosuppression, which is an exceptional occurrence.

Few similar cases have been reported in the literature of disseminated African histoplasmosis with multifocal lesions affecting the skin, bones, and lymph nodes and unusually associated with HIV. Hepatosplenomegaly is due to invasion of the liver and spleen by mycosis, an intracellular parasite infecting the reticuloendothelial phagocytic cells [10,11]. This was confirmed after their regression after itraconazole treatment. The main diagnostic challenge lies in the non-specific symptoms of histoplasmosis, which are often clinically indistinct and can lead to misdiagnosis, particularly in non-endemic areas.

The treatment for moderate disseminated histoplasmosis in immunocompetent patients is oral itraconazole 200 mg twice daily for one year [3]. However, the treatment for moderate disseminated disease in HIV patients is amphotericin B at 3 mg/kg IV for two weeks, followed by itraconazole 200 mg orally three times daily for three days, and then twice daily for one year [12]. Our patient was not immunocompromised and had moderate, non-life-threatening disease, which is why we opted for itraconazole 200 mg twice daily for 12 months. We noted the disappearance of skin lesions after one month of treatment, as well as the regression of splenomegaly and hepatomegaly on follow-up ultrasound. The patient did not show any recurrence after one year of follow-up.

## Conclusions

It is well documented that disseminated histoplasmosis is most common among patients with immunodeficiency; however, immunocompetent patients can also be affected, especially after high inoculum exposure. The nonspecific symptoms of histoplasmosis are often clinically indistinct and may mislead the diagnosis, especially in non-endemic zones. Early diagnosis and treatment of disseminated histoplasmosis are particularly beneficial and contribute to improved outcomes.

This case illustrates the rarity of disseminated histoplasmosis in immunocompetent individuals, particularly in non-endemic regions, and the importance of considering a travel history to endemic regions. Our patient presented a very good response to treatment with itraconazole, which supports the importance of early diagnosis and appropriate therapy.
